# Mechanistic Evaluation of Roxadustat for Pulmonary Fibrosis: Integrating Network Pharmacology, Transcriptomics, and Experimental Validation

**DOI:** 10.3390/ph19010179

**Published:** 2026-01-20

**Authors:** Congcong Zhang, Xinyue Huang, Huina Ye, Haidong Tang, Minwei Huang, Shu Jia, Jingping Shao, Jingyi Wu, Xiaomin Yao

**Affiliations:** College of Pharmacy, Zhejiang Pharmaceutical University, Ningbo 315000, China; zcc1114@126.com (C.Z.);

**Keywords:** pulmonary fibrosis, roxadustat, network pharmacology, transcriptomics, mice

## Abstract

**Background**: Pulmonary fibrosis (PF) currently lacks effective therapeutic interventions. Roxadustat, an oral small-molecule inhibitor of hypoxia-inducible factor prolyl hydroxylase, has been shown in several studies to attenuate the progression of fibrotic diseases. However, its therapeutic efficacy in PF remains to be fully elucidated. The aim of this study was to evaluate roxadustat’s therapeutic benefits on PF as well as the underlying mechanisms of action. **Methods**: Bleomycin was administered intraperitoneally to establish a PF mouse model. H&E staining, Masson staining, and immunohistochemistry (IHC) were used to assess histopathological and fibrotic changes. Changes in the expression levels of inflammatory mediators, including IL-1β, TGF-β1, and TNF-α, were examined by reverse transcription-quantitative polymerase chain reaction (RT-qPCR). Network pharmacology combined with transcriptomic analysis was employed to identify potential target genes and associated signaling pathways. Subsequently, RT-qPCR and Western blot analyses were carried out to experimentally validate the predicted targets and pathways and to verify the protective effects of roxadustat in PF mice. **Results**: Roxadustat markedly ameliorated bleomycin-induced pulmonary fibrosis in mice. The therapeutic effect was evidenced by a reduction in alveolar damage, thinner alveolar septa, diminished infiltration of inflammatory cells, and decreased collagen deposition. Concomitantly, the expression levels of inflammatory mediators, including IL-1β, TGF-β1, and TNF-α, were significantly lowered. Integrated network pharmacology and transcriptomic analyses revealed the involvement of critical signaling pathways, specifically nuclear factor-kappa B (NF-κB) and peroxisome proliferator-activated receptor (PPAR). Experimental validation further demonstrated that roxadustat downregulated the expression of key genes (S100A8, S100A9, and Fos) in murine lung tissues. It also suppressed the protein ratios of phosphorylated p65 to total p65 and phosphorylated IκBα to total IκBα. Moreover, roxadustat treatment upregulated PPARγ protein expression. **Conclusions**: These data indicate that roxadustat ameliorates bleomycin-induced PF in mice, an effect associated with modulation of the NF-κB and PPAR signaling pathways. The findings provide a preclinical rationale for further investigation of roxadustat as a potential treatment for PF.

## 1. Introduction

Pulmonary fibrosis (PF) represents the end-stage manifestation of a diverse spectrum of lung disorders, characterized pathologically by fibroblast proliferation and excessive deposition of extracellular matrix (ECM), along with concomitant inflammatory injury and structural tissue destruction [1]. Idiopathic pulmonary fibrosis (IPF), which constitutes the majority of PF cases, is associated with a poor prognosis, with a median survival of only 3–5 years after diagnosis [2]. While antifibrotic agents such as pirfenidone and nintedanib have demonstrated efficacy in reducing the frequency of acute exacerbations and slowing the decline in lung function among patients with mild-to-moderate disease, their clinical utility is limited by a significant burden of adverse effects. [3,4]. Thus, the development of innovative treatments that can stop the progression of the disease and enhance patient outcomes is desperately needed.

Originally developed for anemia associated with chronic kidney disease, Roxadustat acts as a potent inhibitor of hypoxia-inducible factor prolyl hydroxylase (HIF-PHD). By reversibly inhibiting PHD enzyme activity, it transiently stabilizes hypoxia-inducible factors (HIFs). This stabilization promotes a brief upregulation of HIF-regulated genes, notably erythropoietin (EPO) in the kidneys and liver, ultimately stimulating erythropoiesis and correcting anemic conditions [5]. Emerging studies suggest that roxadustat may also ameliorate hypoxia-related pathologies, such as fibrosis [6], inflammatory response [7], and ferroptosis [8]. Experimental data indicate that roxadustat enhances random flap viability through mechanisms involving augmented vascular growth and reduced concentrations of inflammatory mediators, including IL-1β and TNF-α [9]. In IPF, roxadustat has been shown to attenuate lung injury, likely via modulation of the TGF-β1/Smad signaling pathway [10]. Although roxadustat shows promise for pulmonary fibrosis, its mechanisms remain unclear. Therefore, the application of contemporary scientific methodologies, particularly advanced multi-omics approaches, is crucial to elucidate its mechanistic basis and fully harness its clinical potential.

Network pharmacology, rooted in systems biology and polypharmacology principles, leverages interactive networks linking drugs, targets, diseases, and genes to systematically elucidate multi-target drug actions and pathway modulations within biological systems [11]. Complementing this, Transcriptomics is a widely used technique for investigating disease-target interactions and analyzing the pharmacological mechanisms of drugs at the RNA level, both in vivo and in vitro [12]. The integration of these strategies facilitates a comprehensive dissection of roxadustat’s therapeutic mechanisms in pulmonary fibrosis, revealing potential molecular targets for therapeutic intervention.

Consequently, this study utilized histopathological analysis through H&E and Masson’s trichrome staining, along with IHC. The effects of roxadustat on inflammatory mediators were evaluated using reverse transcription-quantitative polymerase chain reaction (RT-qPCR). By integrating network pharmacology with transcriptomic profiling, potential key targets and pathways of roxadustat in pulmonary fibrosis were identified. Subsequent validation was carried out using RT-qPCR and Western blot analyses. Collectively, this work is expected to provide a theoretical foundation for the application of roxadustat in the treatment of pulmonary fibrosis.

## 2. Result

### 2.1. Roxadustat Improves Lung Tissue Metabolism in Mice with PF

**Figure 1:**
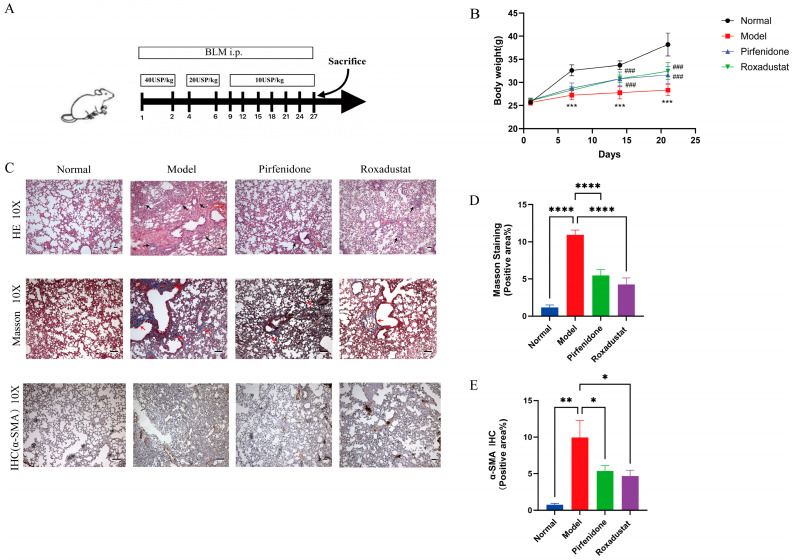
Roxadustat inhibits PF in mice caused by bleomycin. (**A**) Diagram of the PF mice model caused by bleomycin, (**B**) Changes in body weight in experimental groups at specified intervals (*n* = 6), (**C**) The images of H&E (Scale bars: 50 µm, black arrows indicate prominent areas of inflammatory cell infiltration), Masson staining (Scale bars: 100 µm, red arrows indicate prominent areas of collagen deposition) and Immunohistochemical (Scale bars: 100 µm). (**D**) Area statistics of collagen fibers in lung tissue (*n* = 5), (**E**) Quantification of α-SMA-positive area (*n* = 5). Higher-magnification (20×) views are provided in Supplementary Figure S1. (* *p* < 0.05 vs. Normal, ** *p* < 0.01 vs. Normal, *** *p* < 0.001 vs. Normal, **** *p* < 0.0001 vs. Normal, ^###^ *p* < 0.001 vs. Model).

The results showed that mice administered bleomycin exhibited a significant reduction in body weight, whereas treatment with roxadustat or pirfenidone led to an increase in body weight (Figure 1B). Histological analysis revealed that while the Normal group exhibited intact lung architecture, bleomycin administration induced severe alveolar damage, thickening of alveolar walls, inflammatory infiltration and collagen deposition, all of which were significantly ameliorated by roxadustat or Pirfenidone (Figure 1C,D). IHC analysis showed that bleomycin upregulated α-SMA expression, an effect that was significantly reduced following administration of either roxadustat or pirfenidone (Figure 1C,E).

### 2.2. Roxadustat Reduces Inflammation in Mice Lung Tissue

**Figure 2:**
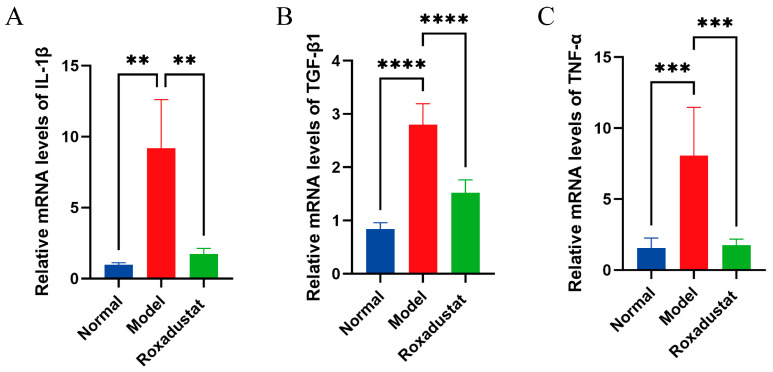
Roxadustat decreased lung tissue inflammation in mice. The relative mRNA expression levels are presented for (**A**) IL-1β (*n* = 6), (**B**) TGF-β1 (*n* = 6), (**C**) TNF-α (*n* = 5). (Data are expressed as the mean ± SD; ** *p* < 0.01, *** *p* < 0.001, **** *p* < 0.0001).

Our investigation revealed a significant contribution of inflammatory processes to pulmonary fibrosis pathogenesis. To evaluate the anti-inflammatory effects of roxadustat, we quantified the expression of pro-inflammatory cytokines in murine lung tissue using RT-qPCR. The fibrotic model showed marked upregulation of IL-1β, TGF-β1, and TNF-α transcripts relative to control animals, whereas administration of roxadustat significantly reduced the expression levels of these cytokines (Figure 2).

### 2.3. Identification of Roxadustat Targets in PF Treatment

**Figure 3:**
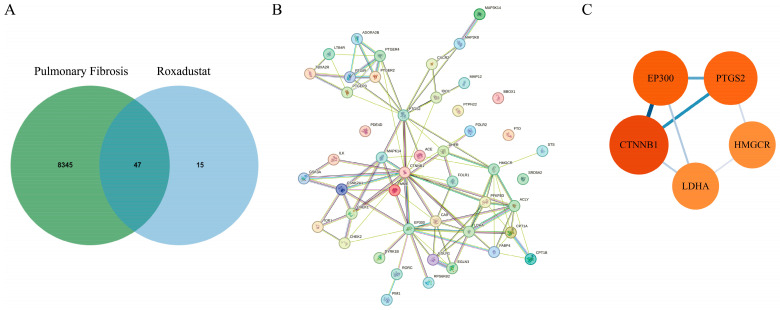
Identification of roxadustat targets in PF treatment. (**A**) PF and roxadustat target gene intersection in the Venn diagram. The intersection reveals 47 common targets between roxadustat and PF. (**B**) PPI network of roxadustat acting on 47 common targets. (**C**) Target genes for roxadustat network diagram. Core targets with high connectivity, suggesting central roles in the therapeutic network, include *CTNNB1*, *PTGS2*, *EP300*, *LDHA*, and *HMGCR*. P_FDR_ < 0.05.

Sixty-two potential target genes were identified from the PharmMapper databases, contrasted with 8392 disease-associated targets collected from GeneCards, OMIM, and TTD. Subsequent intersection analysis revealed 47 overlapping targets that are potentially involved in the anti-pulmonary fibrosis mechanism of roxadustat (Figure 3A). These candidate targets were subjected to PPI network analysis, resulting in a network comprising 47 nodes interconnected by 104 edges (Figure 3B).

The target interactions were systematically analyzed using Cytoscape 3.8.0. Network visualization based on degree centrality (Figure 3C) revealed several core targets, including *CTNNB1, PTGS2, EP300, LDHA, and HMGCR*.

### 2.4. Functional Analysis of Common Targets of Roxadustat in PF Treatment

**Figure 4:**
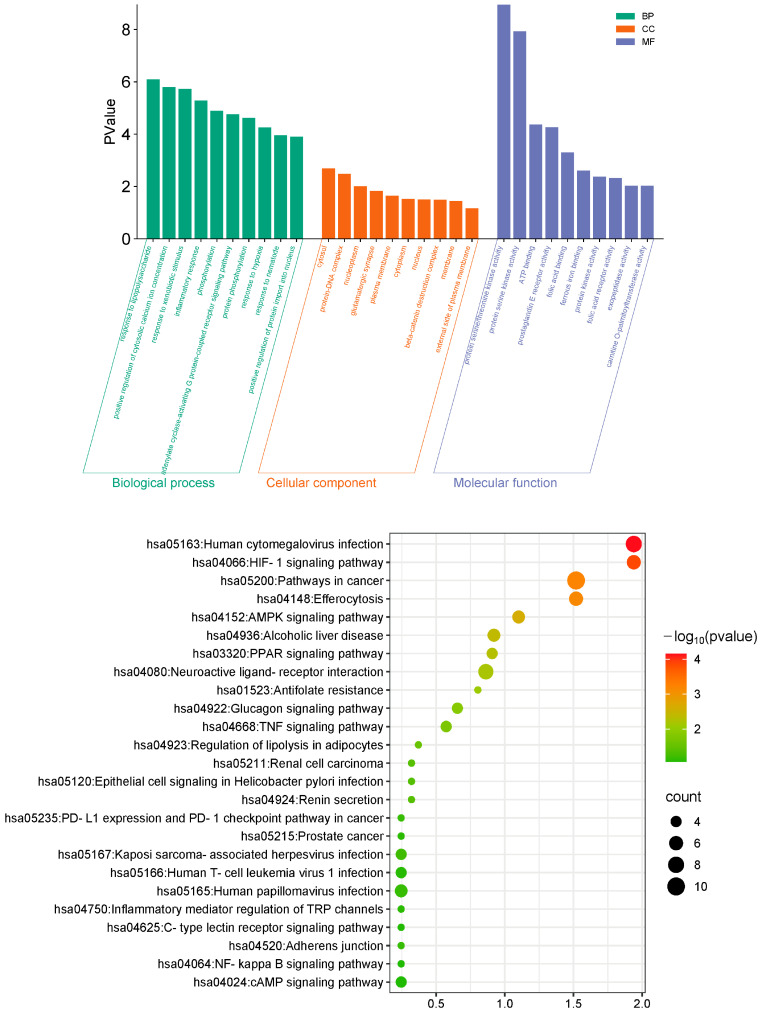
Predicted possible mechanisms of roxadustat in the treatment of PF. (**Top**) GO functional enrichment analysis of roxadustat treatment targets for PF. The biological processes primarily involve response to lipopolysaccharide, regulation of inflammation, and response to xenobiotic stimuli. Cellular components include cytosol and nucleoplasm, and enriched molecular functions encompass serine/threonine kinase activity and ATP binding. (**Bottom**) KEGG pathway enrichment analysis of roxadustat treatment targets for PF. The significantly enriched pathways include NF-κB signaling, PPAR signaling, and AMPK signaling pathways.

GO enrichment analysis revealed that the therapeutic targets of roxadustat in pulmonary fibrosis are primarily involved in key biological pathways, including lipopolysaccharide response, modulation of intracellular calcium levels, regulation of inflammation, and response to xenobiotic compounds. The cellular components include the cytosol, nucleoplasm, glutamatergic synapse, and plasma membrane. Enriched molecular functions encompass serine/threonine-protein kinase activity, activation of prostaglandin receptors, protein kinase catalytic function, and adenosine triphosphate binding (Figure 4, top). KEGG analysis indicated that roxadustat’s therapeutic targets are predominantly enriched in several critical signaling pathways, including HIF signaling, NF-κB, PPAR, and the AMPK signaling pathway (Figure 4, bottom). These results suggest that roxadustat’s anti-fibrotic properties likely involve coordinated modulation across diverse molecular networks and biological pathways.

### 2.5. RNA-Seq Profiling and Differential Gene Expression Analysis

**Figure 5:**
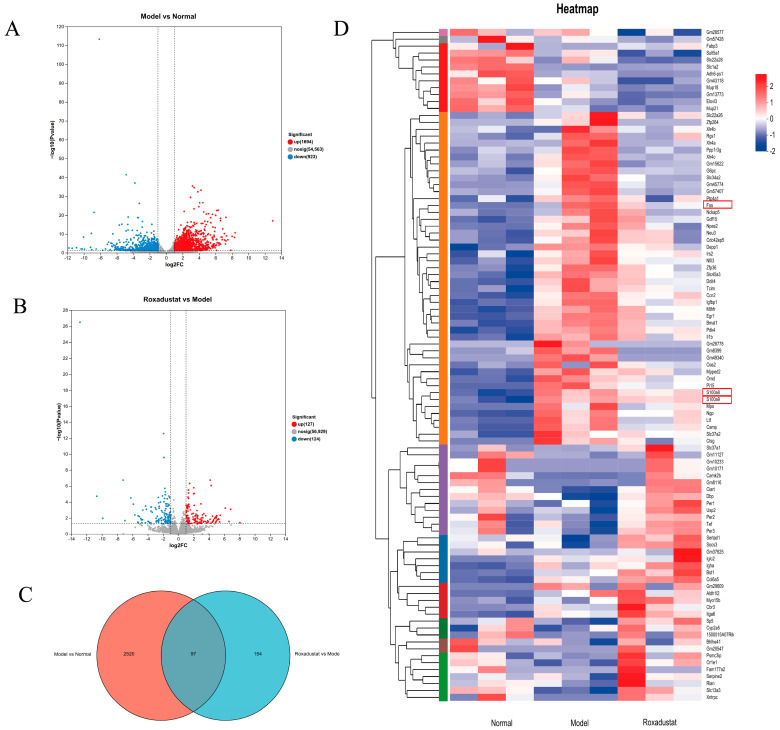
Expression patterns of differently expressed genes. (**A**) Volcano plot of DEGs in Model vs. Normal lungs. Bleomycin-induced PF significantly altered 2617 genes (1694 upregulated, 923 downregulated). (**B**) Volcano plot of DEGs in Roxadustat vs. Model lungs. Treatment significantly modulated 251 genes (127 upregulated, 124 downregulated). (**C**) Venn diagram showing overlap of DEGs between the two comparisons. Roxadustat specifically modulated 97 genes (overlapping set) that were also altered by PF. (**D**) Heatmap displaying the hierarchical clustering of mRNAs in different groups. Representative roxadustat-reversed genes are highlighted by red boxes.(e.g., S100A8, S100A9, and Fos), which were upregulated in Model vs. Normal but downregulated towards normal levels after roxadustat treatment.

Using the DESeq2 package, we identified significantly differentially expressed genes based on the criteria |log2FC| ≥ 1 and *p* < 0.05 after multiple testing correction. Transcriptional changes across the three groups were visualized using volcano plot analysis (Figure 5A). Comparative transcriptomic analysis revealed 2617 differentially expressed genes (DEGs) in the Model group compared to Normal controls (1694 upregulated, 923 downregulated). Roxadustat treatment modulated 251 transcripts (127 upregulated, 124 downregulated). A Venn diagram analysis identified 97 overlapping DEGs, which may represent key mediators of roxadustat’s therapeutic effects in PF (Figure 5B). Heatmap clustering analysis of the DEGs (Figure 5C,D) revealed that genes such as S100 calcium-binding protein A8 (S100A8), A9 (S100A9), and Fos proto-oncogene (Fos) showed distinct expression in the roxadustat group relative to the model group, as well as in the model group relative to the normal group. The full list of these 97 overlapping DEGs is provided in Supplementary Table S1. Key metrics of data quality, including sequencing depth, read mapping statistics, and PCA clustering results, are provided in the Supplementary Materials (Supplementary Tables S2 and S3 and Figure S2).

### 2.6. KEGG Analysis

**Figure 6:**
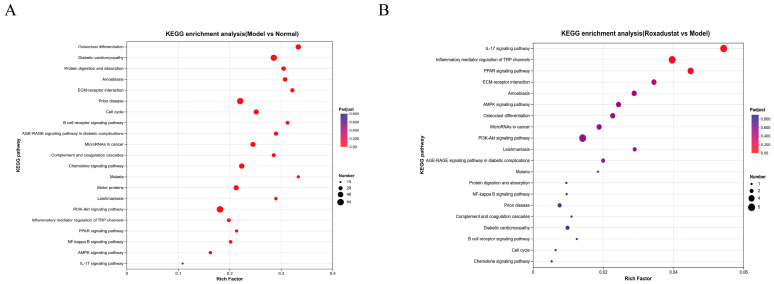
KEGG analysis results of DEGs. (**A**) Model and Normal. Key pathways implicated in PF pathogenesis include ECM-receptor interaction, PI3K-Akt, NF-κB, and PPAR signaling pathways. (**B**) Roxadustat and Model. Treatment notably affected pathways related to inflammation and metabolism, including inflammatory mediator regulation of TRP channels, PPAR, NF-κB, IL-17, and TNF signaling pathways.

The KEGG pathway database was employed to identify the DEGs in the two comparisons. In the model vs. normal comparison, ECM-receptor interaction, osteoclast differentiation, diabetic cardiomyopathy, and the PI3K-Akt, NF-κB, and PPAR signaling pathways were significantly enriched (Figure 6A). Meanwhile, DEGs that were differently expressed in the roxadustat group compared to the Model group were primarily associated with pathways such as inflammatory mediator regulation of TRP channels, ECM-receptor interaction, and the PPAR, NF-κB, IL-17, AMPK, and TNF signaling pathways (Figure 6B). Notably, two important molecular pathways were found to be significantly enriched in both comparisons: PPAR and NF-κB, with these observations showing concordance with prior network pharmacology predictions.

These results suggest that roxadustat may ameliorate PF by modulating the PPAR and NF-κB signaling pathways, potentially through the regulation of key inflammatory mediators, such as S100A8, S100A9, and Fos.

### 2.7. Validation of Gene Expression Profiles

**Figure 7:**
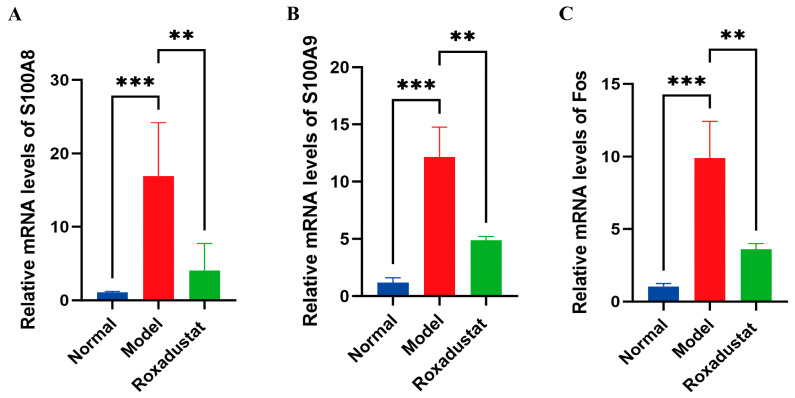
Expression levels of three representative DEGs. (**A**) S100A8 (*n* = 5), (**B**) S100A9 (*n* = 6), (**C**) Fos (*n* = 6). ** *p* < 0.01, *** *p* < 0.001.

In order to confirm the DEGs detected in transcriptomic studies, we conducted RT-qPCR of three roxadustat-related DEGs implicated in PF treatment. These DEGs are primarily associated with the NF-κB and PPAR signaling pathways. As shown in Figure 7, the expression levels of S100A8, S100A9, and Fos were consistent with the transcriptomic data, thus confirming the reliability of the transcriptomic findings.

### 2.8. Roxadustat’s Impact on the NF-κB/PPARγ Pathways

**Figure 8:**
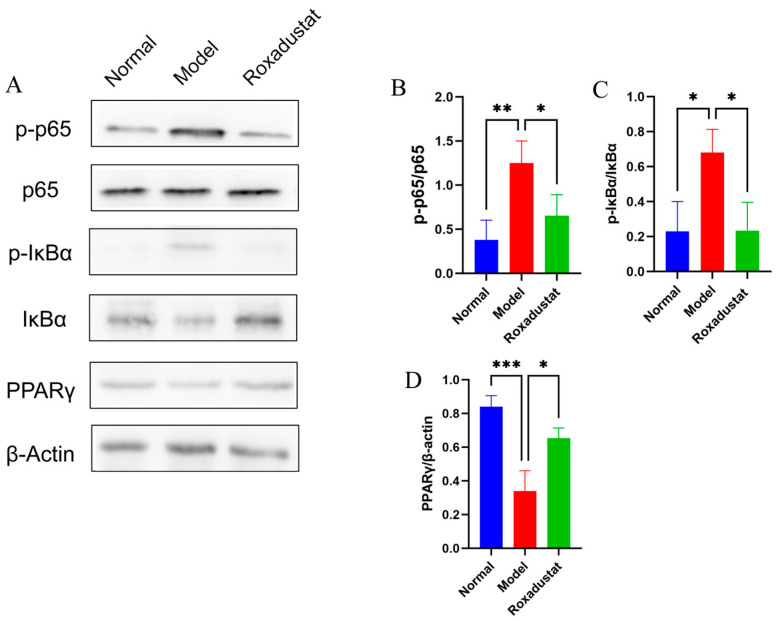
Western blot analysis of the expression of NF-κB and PPARγ signaling pathway-related proteins. (**A**) Individual protein bands and groupings (*n* = 3). (**B**) p-p65/p65 ratio. (**C**) p-IκBα/IκBα ratio. (**D**) PPARγ/β-Actin ratio. * *p* < 0.05, ** *p* < 0.01, *** *p* < 0.001.

Differential expression of PPARγ and NF-κB pathway proteins in lung tissues was assessed by Western blot analysis (Figure 8 and Figure S3). The Model group exhibited markedly reduced PPARγ levels alongside elevated p-p65 and p-IκBα. Treatment with roxadustat markedly reversed these alterations, upregulating PPARγ expression and inhibiting activation of the NF-κB signaling pathway.

## 3. Discussion

PF is characterized as a chronic, refractory, and progressive lung disorder. Its pathogenesis involves multiple pro-fibrotic mediators, which promote the differentiation of fibroblasts into myofibroblasts and drive excessive ECM production [13,14]. These pathological alterations ultimately result in organ dysfunction, impaired gas exchange, and respiratory failure [15,16]. Currently, α-SMA is regarded as a specific marker indicative of myofibroblast activation in this process [17]. In a murine model of pulmonary fibrosis induced by intraperitoneal administration of bleomycin [18], HE staining revealed alveolar structural disruption and inflammatory cell infiltration in lung tissues. Meanwhile, Masson staining showed extensive collagen deposition in the pulmonary interstitium, and IHC assays confirmed elevated expression of α-SMA in the lung parenchyma. These hallmark features collectively confirm the successful establishment of the disease model, consistent with previously reported bleomycin-induced pulmonary pathology.

Pulmonary fibrosis models induced by bleomycin can be established via two primary approaches: localized intratracheal delivery or systemic distribution [19]. The intratracheal method, although capable of directly injuring alveolar epithelial cells, typically results in transient fibrotic changes that resolve within four weeks [20,21,22]. Conversely, systemic administration through intraperitoneal injection provides operational advantages, including simplified technical execution and accurate dosing, while consistently producing persistent fibrotic alterations. Contemporary studies indicate this systemic approach better recapitulates the progressive nature of human pulmonary fibrosis [19,23]. Consequently, our experimental design incorporated an intraperitoneal bleomycin challenge for murine model establishment.

Our research demonstrates that roxadustat significantly ameliorates bleomycin-induced PF in mice by improving pathological alterations in lung tissue, reducing collagen deposition, and suppressing the expression of α-SMA. To elucidate the underlying mechanisms of roxadustat in PF treatment, we employed network pharmacology to clarify pertinent targets and pathways. Network pharmacology analysis identified 47 potential targets of roxadustat in PF treatment, with key targets including *CTNNB1, PTGS2, EP300, LDHA, and HMGCR*. According to the functional enrichment study, these targets are implicated in biological functions such as the regulation of cytosolic calcium ion concentration, inflammatory response, and reaction to xenobiotic stimuli. Furthermore, KEGG pathway analysis highlighted the involvement of several critical signaling pathways, including PPAR, TNF, NF-κB, and IL-17.

Although network pharmacology offers valuable theoretical predictions based on database analyses, it lacks real-time biological verification. To address this limitation, transcriptomic analysis was incorporated [24]. To this end, this approach was implemented to validate the pathway predictions derived from network pharmacology, thereby establishing a robust correlation between computational predictions and experimental biological responses.

Transcriptomic analysis revealed significant alterations in the gene expression profile following roxadustat treatment. Key DEGs, including S100A8, S100A9, and Fos, were identified. S100A8 and S100A9 are calcium-binding proteins involved in inflammatory and immune responses [25], and Fos regulates the expression of inflammatory genes [26]. The transcription factor c-Fos, encoded by the Fos gene, is a central component of the activator protein-1 (AP-1) complex. It is noteworthy that binding sites for AP-1 are present within the promoter regions of both S100A8 and S100A9 genes. This interaction facilitates their transcriptional activation, thereby establishing a functional link between these critical differentially expressed genes [27]. S100A8 and S100A9 are calcium-modulated members of the S100 protein family and are predominantly produced by immature myeloid cells, particularly granulocytic populations [28]. These proteins commonly form heterodimeric complexes (S100A8/A9), which play crucial roles in cytoskeletal organization, maintenance of calcium homeostasis, and modulation of enzymatic activities [29]. Toll-like receptor 4 (TLR4), a critical pattern recognition receptor within the TLR family, serves as a primary receptor for calcium-binding proteins and exhibits a strong functional interdependence with S100A8/A9 [30]. Their interaction initiates downstream signaling cascades, leading to amplified inflammatory responses [31]. In the pathogenesis of PF, inflammatory mechanisms contribute substantially [32]. Elevated levels of S100A8/A9 are released and bind to TLR4, triggering myeloid differentiation primary response 88 (MyD88)-dependent signaling pathways [33]. This leads to phosphorylation of IκBα, resulting in NF-κB activation and subsequent nuclear translocation [34]. Once in the nucleus, NF-κB regulates the transcription of multiple pro-inflammatory mediators, including TNF-α, IL-1β, and TGF-β1 [35]. Consequently, S100A8, S100A9, and Fos were identified as key target genes for further investigation via transcriptomics. In the animal studies, mice with pulmonary fibrosis exhibited significantly dysregulated expression of these genes, which was effectively mitigated following treatment with roxadustat.

Additionally, enrichment of differentially expressed genes (DEGs) in the PPAR and NF-κB pathways supports the hypothesis that roxadustat alleviates fibrosis through modulation of these signaling cascades. PPARγ serves as a key negative regulator of immunological and inflammatory processes. By directly interacting with NF-κB, PPARγ inhibits its nuclear translocation, thereby suppressing NF-κB-mediated signaling [36]. This antagonistic interaction dampens inflammatory activation and exerts cytoprotective effects. The capacity of PPAR agonists to inhibit inflammatory cell infiltration has been well documented in numerous studies, and these agents are commonly employed in clinical practice for the treatment of inflammation-associated diseases [37]. Through the combination of network pharmacology and transcriptomics analysis, the study suggests that the anti-fibrotic mechanism of roxadustat in mice likely involves the regulation of inflammatory pathways, particularly via TNF-α, TGF-β1, IL-1β, NF-κB, and PPAR signaling pathways. In animal tests, roxadustat administration significantly decreased TNF-α, TGF-β1, IL-1β, p-p65/p65, and p-IκBα/IκBα expression levels; however, PPARγ expression was elevated. These findings indicate that roxadustat’s therapeutic benefits in PF are correlated with the suppression of the NF-κB pathway and the activation of the PPAR signaling pathway, which would ultimately lead to therapeutic benefits for PF.

HIF-1α functions as a master regulator in response to hypoxic stimuli. Under normoxic conditions, this subunit is primarily localized in the cytoplasm and subject to rapid proteasomal degradation, resulting in typically undetectable expression levels [38]. During hypoxia, HIF-1α translocates to the nucleus, where it dimerizes with HIF-1β to drive the transcription of downstream target genes, thereby facilitating cellular adaptation to low-oxygen environments [39]. Although the present study did not directly measure HIF-α protein levels, it is relevant to consider its potential role in the observed anti-fibrotic mechanisms of roxadustat, a potent HIF-PHD inhibitor. Accumulating evidence indicates a complex interplay between HIF-1α and the NF-κB/PPARγ pathways. For instance, in a murine periodontitis model, dimethyloxallylglycine (DMOG), an activator of HIF-1α, was shown to elevate HIF-1α expression, suppress phosphorylation of the NF-κB pathway, and downregulate the expression of cytokines such as TNF-α and IL-6 [40]. This suggests that the anti-inflammatory effects of HIF-1α may involve negative regulation of NF-κB signaling. Conversely, stabilization of HIF-1α has also been linked to PPARγ activation [41]. Therefore, as discussed herein, the inhibition of NF-κB and activation of PPARγ associated with roxadustat treatment may correlate with HIF-α stabilization. Future studies should aim to measure HIF-1α expression directly to clarify the precise hierarchical interactions among these signaling pathways.

Here are some limitations of this study. First, although RT-qPCR was employed to quantify the transcriptional levels of pivotal pro-inflammatory mediators, providing initial insights into roxadustat’s anti-inflammatory potential in PF, our study did not include IHC quantification of inflammation rates using specific cellular markers (e.g., CD68 for macrophages). While RT-qPCR allows assessment of the overall inflammatory status in lung tissue, IHC-based analysis could yield valuable spatial information regarding inflammatory cell infiltration, representing an important focus for subsequent investigations. Second, this work is confined to a BLM-induced PF model in mice. Future studies should systematically evaluate the dosing regimen and potential toxicity of roxadustat. Third, the specific contributions of individual targets and pathways identified in this study require further experimental validation. Functional investigations using gene knockout or overexpression models, or cellular gene-silencing models, would make it easier to understand how exactly these targets contribute to roxadustat’s antifibrotic actions [42]. Moreover, it is important to note that the current study did not include pirfenidone as a positive control in evaluating the anti-inflammatory efficacy and underlying mechanisms of roxadustat. Pirfenidone is known to exert its anti-fibrotic effects largely via suppression of TGF-β1 signaling [43]. In contrast, our findings suggest that roxadustat, a potent HIF-PHD inhibitor, may modulate PF through pathways involving NF-κB and PPAR signaling. Given the distinct mechanistic underpinnings of these two agents, direct comparative analyses would demand more rigorous experimental design; however, resource limitations prevented the full implementation of this objective in the present study. Future studies will specifically incorporate pirfenidone as a reference control to enable a direct head-to-head comparison of the anti-inflammatory and anti-fibrotic efficacy between roxadustat and this established clinical agent. This subsequent work is crucial for clarifying the potential therapeutic positioning of roxadustat in the management of PF. Furthermore, the sample size for some quantitative analyses, particularly Western blotting (*n* = 3), while consistent with initial exploratory studies, may benefit from expansion in future confirmatory research to enhance statistical power and generalizability.

## 4. Materials and Methods

### 4.1. Animal Experiment

Twenty-four 8-week-old male C57BL/6J mice (18–22 g) were obtained from the Zhejiang Academy of Medical Sciences (Hangzhou, China). All animal experiments were conducted in compliance with the Animal Research Reporting of In Vivo Experiments (ARRIVE) guidelines and were approved by the Institutional Animal Care and Use Committee of Zhejiang Pharmaceutical University (Approval No. 202502002). Throughout the study, all efforts were made to minimize animal suffering and ensure their welfare.

Mice were randomly allocated to the four experimental groups using a computer-generated random number sequence: (1) Normal group (*n* = 6), (2) Model group (*n* = 6), (3) Pirfenidone group (*n* = 6), and (4) Roxadustat group (*n* = 6). On days 1 and 2, mice in the model, pirfenidone (GC12790, GlpBio, Montclair, CA, USA), and roxadustat (purity > 98%, C19H16N2O5, CAS No. 808118-40-3, Biochempartner, Shanghai, China) groups received intraperitoneal injections of bleomycin (Shanghai Yuanye Bio-Technology Co. Ltd., Shanghai, China; 40 USP/kg). From experimental days 4 to 27 [44,45], bleomycin was delivered in a descending dosage pattern: 20 USP/kg (days 4 and 6) to 10 USP/kg (administered every third day from day 9 to 27) to minimize mortality (Figure 1A). Throughout the experimental period (days 1–27), normal animals received daily intraperitoneal injections of saline (0.2 mL), while treatment groups received once-daily oral gavage of either 50 mg/kg pirfenidone or 30 mg/kg roxadustat. All animals survived until the end of the study and were humanely euthanized by cervical dislocation at termination (Figure 1A).

To preserve blinding, only personnel responsible for compound administration (roxadustat, bleomycin, pirfenidone) had access to group information. All subsequent analytical work was performed by blinded investigators, and group identities remained concealed until the entire dataset was analyzed.

### 4.2. Histopathology

Excised pulmonary specimens were fixed in 4% neutral buffered formalin and subsequently processed for standard paraffin embedding. Serial sections were cut at 5 μm thickness, with subsequent histological processing including both H&E staining and Masson staining (Catalog No. G1340-7, Solarbio, Beijing, China). Collagen deposition was quantified using ImageJ (Version 1.54, National Institutes of Health, Bethesda, MD, USA ) for subsequent statistical evaluation [46].

### 4.3. Immunohistochemistry (IHC)

Following dewaxing, the sections were submerged in 3% H_2_O_2_ for 10 min and then steeped in sodium citrate buffer (0.01 mol/L). The sections were heated in a microwave until boiling, followed by a 7-minute incubation at reduced temperature; this antigen retrieval step was repeated once. Subsequently, sections were blocked with blocking buffer for 20 min at room temperature to minimize non-specific binding. Immunostaining was carried out using Alpha-Smooth Muscle Actin (α-SMA) primary antibody (1:1500, Catalog No. 14395-1-AP, Wuhan Proteintech, Wuhan, China) with overnight incubation at 4 °C. Following thorough washing with phosphate-buffered saline (PBS), HRP-conjugated secondary antibodies were applied for 1 h at room temperature. Antigen detection was performed using 3,3′-diaminobenzidine (DAB) as the chromogen. Digital image analysis for quantification was performed using ImageJ software.

### 4.4. Network Pharmacology

#### 4.4.1. Compilation of Potential Roxadustat Targets

To identify potential molecular targets of roxadustat, we first obtained its standardized Simplified Molecular Input Line Entry System (SMILES) structure from the PubChem database (https://pubchem.ncbi.nlm.nih.gov/, accessed on 4 June 2025 ). This SMILES string was subsequently submitted to the SwissTargetPrediction web tool [47] (http://www.swisstargetprediction.ch/, accessed on 5 June 2025) to predict putative targets. For the purpose of target selection, we set a threshold of probability scores greater than 0 to filter the predicted outcomes. In parallel, the two-dimensional (2D) structural data of roxadustat in SDF format were retrieved from PubChem and uploaded to the PharmMapper database [48] (http://www.lilab-ecust.cn/pharmmapper/, accessed on 5 June 2025) to identify additional target candidates. The predicted targets from both platforms were then integrated, and duplicate entries were removed to generate a non-redundant, comprehensive list of probable molecular targets of roxadustat.

#### 4.4.2. Collection of Gene Targets for PF

To systematically identify genes associated with PF, comprehensive searches were conducted across three major biomedical databases: the Therapeutic Target Database (TTD; https://db.idrblab.net/ttd/, accessed on 7 June 2025), OMIM (https://omim.org/, accessed on 7 June 2025), and GeneCards (https://www.genecards.org/, accessed on 7 June 2025), using the term “Pulmonary Fibrosis” as the primary search query. The resulting potential targets were then standardized to official gene nomenclature through the UniProt knowledgebase (https://www.uniprot.org/, accessed on 8 June 2025) to ensure consistency in subsequent analyses [49].

#### 4.4.3. Identify Potential Therapeutic Targets

Using the Venny 2.1 online platform (http://bioinfogp.cnb.csic.es/tools/venny/, accessed on 9 June 2025), we performed intersection analysis of roxadustat’s potential targets and PF-associated targets, followed by visual representation in Venn diagram format.

#### 4.4.4. Integrated Bioinformatics Analysis of Shared Molecular Targets and PPI Networks

For target discovery in PF, we utilized the STRING platform (https://string-db.org/, accessed on 9 June 2025) to construct a comprehensive network of human proteins involved in key biomolecular interactions. A high-confidence interaction score threshold of 0.4 was applied to ensure the reliability of the predicted associations [50]. The filtered protein–protein interaction (PPI) data were exported in TSV format and subsequently imported into Cytoscape (v3.8.0) for network visualization and graphical representation of the interprotein relationships. This PPI network provides insights into the complex biomolecular interactions relevant to PF treatment. Network topological features were computationally assessed using CytoNCA to determine the key targets of roxadustat in pulmonary fibrosis treatment. Critical metrics such as node degree, betweenness centrality, closeness centrality, and average shortest path length were analyzed, followed by degree-based ranking to pinpoint the most influential nodes in the network.

#### 4.4.5. Functional Enrichment Analysis

The Metascape platform (https://metascape.org/, accessed on 10 June 2025) was employed to perform comprehensive enrichment studies of Gene Ontology (GO) keywords and Kyoto Encyclopedia of Genes and Genomes (KEGG) pathways, aiming to identify potential therapeutic targets. Comprehensive functional characterization included all three Gene Ontology (GO) domains (BP, CC, MF) in addition to Kyoto Encyclopedia of Genes and Genomes (KEGG) pathway analysis. Analytical results were systematically extracted and visualized to provide insights into the functional roles and pathway associations of the target genes.

### 4.5. Analysis of Transcriptomes

#### 4.5.1. Sequencing and Separation of RNA

In accordance with the manufacturer’s instructions, lung tissues were dissolved in TRIzol^®^ Reagent for RNA isolation. RNA integrity was assessed using an Agilent 5300 Bioanalyzer (Santa Clara, CA, USA), while concentration measurements were performed with a NanoDrop ND-2000 spectrophotometer (Waltham, MA, USA). For transcriptome library construction, 1 μg of total RNA was used with the Illumina^®^ Stranded mRNA Prep kit (San Diego, CA, USA), with mRNA enrichment achieved through polyA+ selection via oligo (dT) magnetic beads. The cDNA library was generated through sequential enzymatic treatments (end-repair, phosphorylation, and adapter ligation). Agarose gel electrophoresis (2% Low Range Ultra) enabled size selection of ~300 bp fragments, followed by limited-cycle amplification (15 cycles) using NEB’s Phusion DNA polymerase (Ipswich, MA, USA). After quantification on a Qubit 4.0 Fluorometer, libraries were subjected to 150 bp paired-end sequencing on an Illumina NovaSeq X Plus system with NovaSeq sequencing chemistry (San Diego, CA, USA).

#### 4.5.2. Gene Expression Variation Analysis and Biological Function Enrichment

Differential gene expression analysis was performed using DESeq2, with significance set at |log2FoldChange| ≥ 1 and *p*-value < 0.05. To further characterize the differentially expressed genes (DEGs), we conducted GO and KEGG pathway enrichment analyses. Significantly enriched GO terms and KEGG pathways were identified based on the whole transcriptome as the background reference, with statistical significance set at a Bonferroni-corrected *p*-value < 0.05. GO enrichment analysis was carried out using Goatools (https://github.com/tanghaibao/GOatools, accessed on 26 July 2025 ), while KEGG pathway enrichment was performed using Python SciPy (https://scipy.org/install/, accessed on 27 July 2025 ).

### 4.6. Real-Time Reverse Transcription–Quantitative Polymerase Chain Reaction (RT-qPCR) Analyses

Total RNA was extracted from lung specimens using a commercial isolation reagent (Vazyme, R701, Nanjing, China), followed by cDNA synthesis with an all-in-one reverse transcription system (Vazyme, R333). RT-qPCR was performed using a SYBR Green-based master mix (Vazyme, Q421-02) on a Roche LightCycler 480 platform under standardized conditions. β-Actin was used as the internal reference gene for normalization. The quantification of gene expression levels was achieved using the 2^−ΔΔCT^ method, with the specific primer sequences employed in this study listed in Table 1.

### 4.7. Western Blot Analyses

Pulmonary tissue homogenates were prepared using RIPA buffer (Bioshap, BL509A, Beijing, China) followed by centrifugation (12,000× *g*, 4 °C) to obtain soluble protein fractions. Protein concentrations were determined using a BCA assay kit (Bioshap, BL521A), and 50 μg of each sample was loaded and separated by 10% SDS-PAGE, then electrotransferred onto PVDF membranes (Merck, ISEQ00010, Darmstadt, Germany). The membranes were then incubated with the following primary antibodies overnight at 4 °C: IkB alpha (380682, Zen-bioscience, Chengdu, China, 1:1000), Phospho-lkB alpha (Ser32/Ser36, 340776, Zen-bioscience, Chengdu, China, 1:1000), NFKB-p65 (phospho Ser536, IPH0008, baijia, Jiangsu, China, 1:1000), NF-KB p65 antibody (80979-1, Proteintech, Wuhan, China, 1:10,000), PPARγ (bs-0530r, Bioss, Beijing, China, 1:1000), and β-Actin (60008-1-Ig, Proteintech, Wuhan, China, 1:5000). After washing, membranes were incubated with HRP-coupled secondary antibody (SA00001-2, SA00001-1, Proteintech, Wuhan, China, 1:5000) and detected with ECL reagent. Images were captured using fluorescent gel imaging equipment (Amersham imager 680; GE, Tokyo, Japan), and Western blotting results were visualized and quantified using ImageJ software. The target protein level was normalized to β-actin. All Western blot comparisons were performed using aliquots from identical sample lysates, with parallel electrophoresis and immunoblotting carried out under consistent experimental conditions.

### 4.8. Statistical Analyses

Statistical analyses in this study were performed using GraphPad Prism 10.0. All experimental data are presented as the mean ± standard deviation (SD) and were derived from at least three independent replicates. Prior to group comparisons, the normality of data distribution was evaluated with the Shapiro–Wilk test, and the homogeneity of variances was evaluated using the Brown-Forsythe test. Based on these assessments, a one-way ANOVA (for equal variances) followed by Bonferroni’s post hoc test for pairwise comparisons, or Welch’s ANOVA (for unequal variances) with an appropriate post hoc test, was applied. *p* < 0.05 was considered to be statistically significant for all statistical analyses.

## 5. Conclusions

Our results indicate that roxadustat treatment ameliorates core pathological manifestations of PF in mice, as evidenced by histological and immunohistochemical analyses, and reduces the levels of inflammatory mediators. Integrated computational and transcriptomic analyses identified S100A8, S100A9, and Fos as key targets and revealed significant involvement of the NF-κB and PPAR signaling pathways. Molecular validation confirmed the downregulation of these targets and a corresponding shift in pathway activity—suppression of NF-κB and promotion of PPAR signaling. Thus, the protective effect of roxadustat appears to be mediated through modulation of this specific gene-pathway network. Further causal validation remains an essential next step. In conclusion, these findings provide a strong preclinical rationale for advancing roxadustat as a potential therapeutic candidate for PF.

## Figures and Tables

**Table 1:** Primer Sequences for qPCR.

Gene	Primer Sequences	GenBank^TM^ Accession No.
β-Actin	Forward AGGCATTGCTGACAGGATG	NM_133360
	Reverse TGCTGATCCACATCTGCTGG	
TNF-α	Forward GAGAAGAGGCTGAGACATAG	NM_001278601.1
	Reverse GTGGAACTGGCAGAAGAG	
IL-1β	Forward TTCTCCACAGCCACAATG	NM_008361.4
	Reverse CAGCAGCACATCAACAAG	
TGF-β1	Forward CTGTATTCCGTCTCCTTGG	NM_011577.2
	Reverse ATTCCTGGCGTTACCTTG	
S100A8	Forward TACTCCTTGTGGCTGTCT	NM_013650.2
	Reverse TTCCTTGCGATGGTGATAA	
S100A9	Forward TGTCCTTCCTTCCTAGAGTAT	NM_001281852.1
	Reverse GCAGCATAACCACCATCA	
Fos	Forward GCAACGCAGACTTCTCAT	NM_010234.3
	Reverse GCTGACAGATACACTCCAA	

## Data Availability

The original contributions presented in this study are included in the article/Supplementary Material. Further inquiries can be directed to the corresponding author. The gene sequencing and processing data reported in this article are stored in the Genome Sequence Archive (https://ngdc.cncb.ac.cn/, accessed on 21 November 2025) under the accession number CRA034140.

## Supplementary Materials

The following supporting information can be downloaded at: https://www.mdpi.com/article/10.3390/ph19010179/s1, Table S1. The 97 overlapping DEGs identified by Venn analysis. Table S2. Sequencing depth of each sample. Table S3. read mapping. Figure S1. The images of H&E (Scale bars: 50 µm, black arrows indicate prominent areas of inflammatory cell infiltration) and Immunohistochemical (Scale bars: 100 µm). Figure S2. PCA mapping of Normal, Model, and Roxadustat samples. Figure S3. The original image of Figure 8.

## Abbreviations

The following abbreviations are used in this manuscript:

Fos	Fos Proto-Oncogene
GO	Gene Ontology
IHC	immunohistochemistry
IL-1β	Interleukin-1β
IL-6	Interleukin-6
IPF	Idiopathic pulmonary fibrosis
KEGG	Kyoto Encyclopedia of Genes and Genomes
NF-κB	Nuclear factor kappa-B
PF	Pulmonary fibrosis
PPARγ	Peroxisome Proliferator-Activated Receptor γ
S100A8	S100 calcium-binding protein A8
S100A9	S100 calcium-binding protein A9
TGF-β1	Transforming Growth Factor-β1
TNF-α	Tumor Necrosis Factor-α
