# Supplementary material for: Mechanistic Evaluation of Roxadustat for Pulmonary Fibrosis: Integrating Network Pharmacology, Transcriptomics, and Experimental Validation
Source: Pharmaceuticals (Basel). 2026 Jan 20;19(1):179. doi: 10.3390/ph19010179 (PMC12844976; doi:10.3390/ph19010179)
Supplement: Supplementary file 1 [file pharmaceuticals-19-00179-s001.zip › pharmaceuticals-4049492-supplementary.pdf]

Table S1. The 97 overlapping DEGs identified by Venn analysis

Gene id	Gene name
ENSMUSG00000000739	Sult5a1
ENSMUSG00000005089	Slc1a2
ENSMUSG00000005547	Cyp2a5
ENSMUSG00000008384	Sertad1
ENSMUSG00000009350	Mpo
ENSMUSG00000016386	Mpped2
ENSMUSG00000018459	Slc13a3
ENSMUSG00000019303	Psmc3ip
ENSMUSG00000019577	Pdk4
ENSMUSG00000019997	Ccn2
ENSMUSG00000020108	Ddit4
ENSMUSG00000020256	Aldh1l2
ENSMUSG00000020429	Igfbp1
ENSMUSG00000020893	Per1
ENSMUSG00000021250	Fos
ENSMUSG00000022389	Tef
ENSMUSG00000022947	Cbr3
ENSMUSG00000024036	Slc37a1
ENSMUSG00000026064	Ptp4a1
ENSMUSG00000026077	Npas2
ENSMUSG00000026249	Serpine2
ENSMUSG00000026358	Rgs1
ENSMUSG00000026435	Slc45a3
ENSMUSG00000027111	Itga6
ENSMUSG00000027398	Il1b
ENSMUSG00000028773	Fabp3
ENSMUSG00000028957	Per3
ENSMUSG00000029009	Mthfr
ENSMUSG00000029082	Bst1
ENSMUSG00000029188	Slc34a2
ENSMUSG00000030256	Bhlhe41
ENSMUSG00000031362	Xlr4c
ENSMUSG00000032010	Usp2
ENSMUSG00000032122	Slc37a2
ENSMUSG00000032484	Ngp
ENSMUSG00000032496	Ltf
ENSMUSG00000032690	Oas2
ENSMUSG00000034427	Myo15b
ENSMUSG00000035239	Neu3
ENSMUSG00000038357	Camp
ENSMUSG00000038418	Egr1
ENSMUSG00000038508	Gdf15

ENSMUSG00000038550	Ciart
ENSMUSG00000038754	Elovl3
ENSMUSG00000038894	Irs2
ENSMUSG00000040314	Ctsg
ENSMUSG00000043889	Gm8399
ENSMUSG00000044786	Zfp36
ENSMUSG00000048368	Omd
ENSMUSG00000048489	Depp1
ENSMUSG00000049690	Nckap5
ENSMUSG00000050423	Ppp1r3g
ENSMUSG00000053113	Socs3
ENSMUSG00000053303	Slc22a26
ENSMUSG00000055116	Bmal1
ENSMUSG00000055866	Per2
ENSMUSG00000056054	S100a8
ENSMUSG00000056071	S100a9
ENSMUSG00000056313	Tcim
ENSMUSG00000056749	Nfil3
ENSMUSG00000057897	Camk2b
ENSMUSG00000059422	Gm8116
ENSMUSG00000059824	Dbp
ENSMUSG00000062128	Or1e1
ENSMUSG00000063590	Slc22a28
ENSMUSG00000063838	Cdc42ep5
ENSMUSG00000066153	Mup21
ENSMUSG00000067768	Xlr4b
ENSMUSG00000067780	Pi15
ENSMUSG00000068165	Gm10233
ENSMUSG00000069825	Gm49340
ENSMUSG00000070425	Xntrpc
ENSMUSG00000075304	Sp5
ENSMUSG00000076937	Iglc2
ENSMUSG00000078650	G6pc
ENSMUSG00000078674	Mup18
ENSMUSG00000079492	Gm11127
ENSMUSG00000079845	Xlr4a
ENSMUSG00000085834	Gm15622
ENSMUSG00000086253	Gm13773
ENSMUSG00000089803	Gm10171
ENSMUSG00000091345	Col6a5
ENSMUSG00000092511	Gm20547
ENSMUSG00000093753	Gm28577
ENSMUSG00000094103	Fam177a2
ENSMUSG00000095079	Igha

ENSMUSG00000097451	Rian
ENSMUSG00000098702	1500015A07Rik
ENSMUSG00000100679	Gm28778
ENSMUSG00000101678	Gm29609
ENSMUSG00000102782	Gm37625
ENSMUSG00000104559	Gm43118
ENSMUSG00000110588	Gm45774
ENSMUSG00000120739	Gm57428
ENSMUSG00000121249	Gm57407
ENSMUSG00000121383	Zfp264
ENSMUSG00000121473	Adh6-ps1

Table S2: Sequencing depth of each sample

sample_name	mean_depth
Normal1	11.6105
Normal2	11.9064
Normal3	16.7461
Model1	10.9429
Model2	10.6777
Model3	10.549
Roxadustat1	9.87018
Roxadustat2	11.4442
Roxadustat3	10.2659

Table S3: read mapping

Sample	Total reads	Total mapped
Normal1	42692428	39241408(91.92%)
Normal2	48189834	44828578(93.02%)
Normal3	45078178	39372994(87.34%)
Model1	46650340	45130523(96.74%)
Model2	44955282	42905842(95.44%)
Model3	44374176	42584027(95.97%)
Roxadustat1	45070050	43638199(96.82%)
Roxadustat2	47860168	46137919(96.4%)
Roxadustat3	43485014	41786677(96.09%)

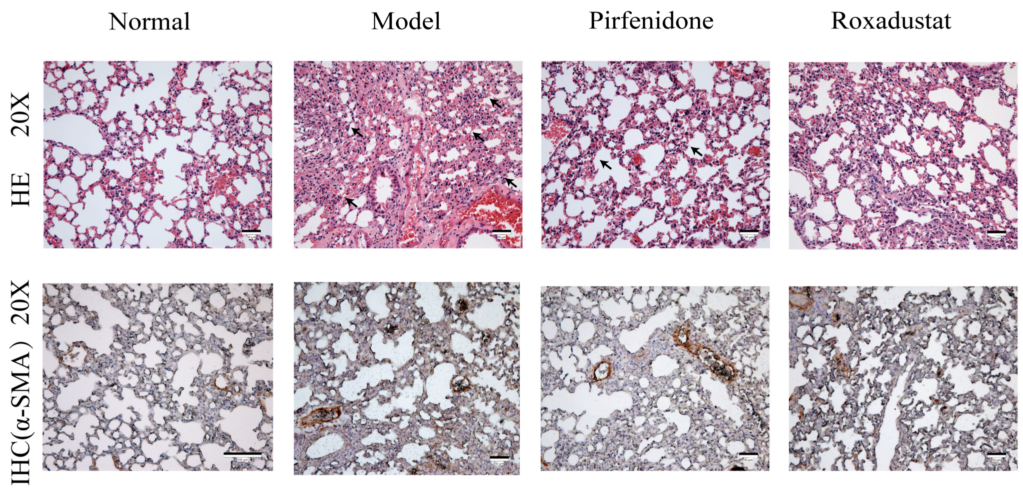

Figure S1. The images of H&E(Scale bars:50 μm, black arrows indicate prominent areas of inflammatory cell infiltration.), and Immunohistochemical (Scale bars:100 μm).

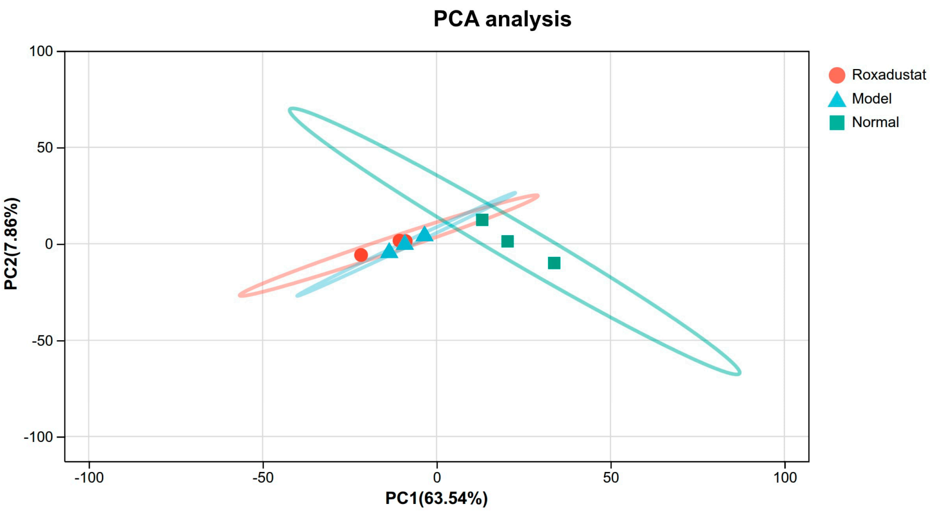

Figure S2. PCA mapping of Normal, Model, and Roxadustat samples.

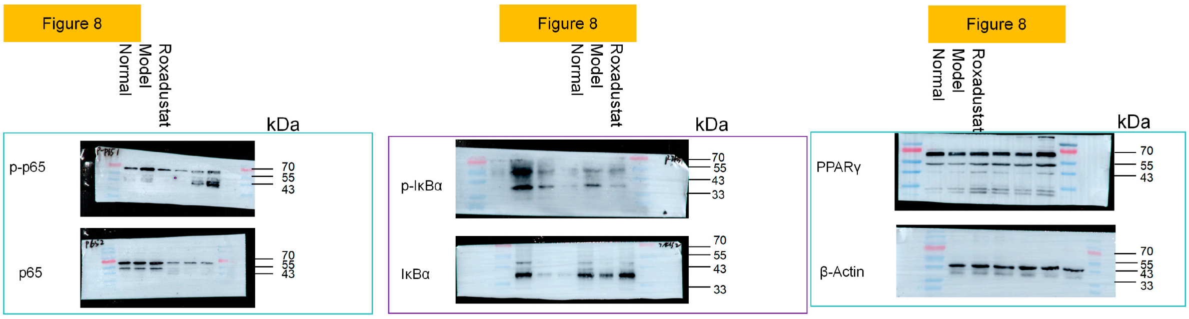

Figure S3. The original image of Figure 8.
